# Sub-MIC Tylosin Inhibits *Streptococcus suis* Biofilm Formation and Results in Differential Protein Expression

**DOI:** 10.3389/fmicb.2016.00384

**Published:** 2016-03-30

**Authors:** Shuai Wang, Yanbei Yang, Yulin Zhao, Honghai Zhao, Jingwen Bai, Jianqing Chen, Yonghui Zhou, Chang Wang, Yanhua Li

**Affiliations:** ^1^Department of Veterinary Pharmacy, College of Veterinary Medicine, Northeast Agricultural UniversityHarbin, China; ^2^Department of Biotechnology, Heilongjiang Vocational College for NationalitiesHarbin, China

**Keywords:** proteomics, *S. suis*, biofilms, tylosin, iTRAQ

## Abstract

*Streptococcus suis* (*S.*suis) is an important zoonotic pathogen that causes severe diseases in humans and pigs. Biofilms of *S. suis* can induce persistent infections that are difficult to treat. In this study, the effect of tylosin on biofilm formation of *S. suis* was investigated. 1/2 minimal inhibitory concentration (MIC) and 1/4 MIC of tylosin were shown to inhibit *S. suis* biofilm formation *in vitro*. By using the iTRAQ strategy, we compared the protein expression profiles of *S. suis* grown with sub-MIC tylosin treatment and with no treatment. A total of 1501 proteins were identified by iTRAQ. Ninety-six differentially expressed proteins were identified (Ratio > ±1.5, *p* < 0.05). Several metabolism proteins (such as phosphoglycerate kinase) and surface proteins (such as ABC transporter proteins) were found to be involved in biofilm formation. Our results indicated that *S. suis* metabolic regulation, cell surface proteins, and virulence proteins appear to be of importance in biofilm growth with sub-MIC tylosin treatment. Thus, our data revealed the rough regulation of biofilm formation that may provide a foundation for future research into mechanisms and targets.

## Introduction

*Streptococcus suis* (*S. suis*) is an important zoonotic pathogen that causes a wide range of diseases in pigs, including meningitis, septicaemia, pneumonia, endocarditis, and arthritis (Gottschalk et al., 2010). In addition, this pathogen is an emerging zoonotic agent and an important public health issue in East and Southeast Asia (Sriskandan and Slater, 2006; Gottschalk et al., 2007). During a single outbreak in China in 2005, more than 200 human cases of *S. suis* were reported, with a death total of 39 (Yang et al., 2006). Current studies show that *S. suis* can cause persistent infections by forming biofilms *in vivo* (Wang et al., 2011).

Biofilms are assemblages of microorganisms characterized by cells that are irreversibly attached to a substratum and embedded in a matrix of self-produced extracellular polymeric substances such as exopolysaccharides (EPS), proteins, nucleic acids and other substances; this type of sessile community-based existence is a critical characteristic for bacterial persistence (Davey and O'Toole, 2000). The physical and biological properties of the biofilm, such as slow growth and mechanical barrier, have a substantial role in the development of increased antimicrobial tolerance. Because the bacteria in chronic infections are aggregated and are in close proximity, genes coding for resistance to antimicrobials can be passed horizontally from one bacterium to the another (Bjarnsholt et al., 2013). The bacteria in biofilms could be 1000-times more difficult to kill by antibiotics than the same organism growing planktonically (Gilbert et al., 1997). Therefore, the control of biofilms is understood to be crucial.

Apart from surgical intervention (when applicable), antibiotics are the main option for the treatment of biofilm infections (Bjarnsholt et al., 2013). Previous studies showed that macrolides successfully inhibited *Staphylococcus aureus* biofilm formation and reduced its virulence when used at sub-inhibitory concentrations (Fujimura et al., 2008). In addition, a sub-minimal inhibitory concentration of erythromycin can inhibit *S. suis* biofilm formation (Zhao et al., 2015). Tylosin, a macrolide antibiotic produced by *Streptomyces fradiae*, is widely used as a veterinary medicine. However, there is still not much effective research of sub-MIC tylosin inhibiting biofilm formation of *S. suis in vitro*.

At present, most of the available information regarding biofilm formation by drug intervention is based on transcriptomic analyses. However, a limitation of transcriptomic analysis for identifying biofilm-regulated gene network raised concerns among investigators. The method of proteomics is thought to be an essential complement to transcriptomic analysis for discovering key regulators of biofilm (Sauer, 2003). Different immunogenic components of planktonically grown *S. suis* proteins such as secreted or cell wall-associated proteins had been studied by using immunoproteomic assays (Zhang and Lu, 2007a,b; Geng et al., 2008; Zhang et al., 2008). Additionally, our lab found that quorum-sensing played a crucial role leading to biofilm formation through quantitative proteomic analysis of *S. suis* biofilm inhibited by sub-MIC erythromycin treatment *in vitro* (Zhao et al., 2015). However, there are no reports regarding the proteomic analysis of sub-MIC tylosin inhibiting biofilm formation of *S. suis in vitro*.

We identified several proteins in sub-MIC tylosin inhibiting biofilm formation of *S. suis* by using iTRAQ technology in this study. The findings from the present study may provide a theoretical foundation for therapy of *S. suis* biofilm infection and provide references for finding new potential therapeutic targets.

## Materials and methods

### Growth of *S. suis* planktonic cells

*S. suis* (ATCC 700794) was grown in Todd-Hewitt yeast Broth (THB; Summus Ltd., Harbin, Heilongjiang, China) for 16–18 h at 37°C with constant shaking for biofilm assays (Wang et al., 2011).

### Observation by scanning electron microscopy (SEM)

Mid-exponential growth phase cultures of *S. suis* ATCC 700794 were adjusted to an optical density of 0.1 at 600 nm (OD600). Then, 2 mL cultures were transferred to the wells of a 6-well microplate containing an 11 × 11 mm sterilized rough glass slide (Mosutech Co., Ltd., Shanghai, China) on the bottom. After culturing for 72 h at 37°C without shaking, the glass slide was removed with tweezers, and the biofilms on the rough glass slide were washed with sterile PBS. The remaining biofilms were fixed with fixative solution [4% (w/v) paraformaldehyde, 2.5% (w/v) glutaraldehyde, 2 mM CaCl_2_ in 0.2 M cacodylate buffer, pH 7.2] for 6 h and washed three times with 0.1 M PBS 10 min each, then fixed in 2% osmium tetroxide containing 2 mM potassium ferrocyanide and 6% (w/v) sucrose in cacodylate buffer. The samples were dried, gold sputtered with an ion sputtering instrument (current 15 mA, 2 min) and observed using SEM (FEI Quanta, Netherland).

### Effect of tylosin on biofilm formation determined by the TCP assay

Mid-exponential growth phase cultures of *S. suis* were adjusted to 0.2 of OD600. Then, 100 μL of cultures were added to each wells of a 96-well microplate with equal volume of tylosin solution with the final concentrations of 1/2 MIC (0.25 μg/mL), 1/4 MIC (0.125 μg/mL), 1/8 MIC (0.0625 μg/mL), and 1/16 MIC (0.03125 μg/mL), respectively. In addition, a negative control (with THB alone) and a positive control (with bacteria alone) were also included. After incubation at 37°C for 72 h without shaking, the medium was removed by aspiration and the wells were washed three times with sterile physiological saline. The remaining attached bacteria were fixed with 200 μL of 99% methanol (Guoyao Ltd., China) per well, and the plates were emptied after 15 min and left to dry. Then, the plates were stained for 5 min with 200 μL of 2% crystal violet (Guoyao Ltd., China) per well. The excess stain was rinsed off by placing the plate under running tap water. After the plates were air dried, the dye bound to the adherent cells was resolubilized with 200 μL of 33% (v/v) glacial acetic acid (Guoyao Ltd., China) per well. The amount of released stain was quantified by measuring the absorbance at 570 nm with a microplate reader (DG5033A, Huadong Ltd., Nanjing, Jiangsu, China). The reported values are the means of three measurements. The experiments were performed in triplicate.

### Colony forming unit (CFU) enumeration

Overnight cultures of *S. suis* were adjusted to an OD 600 of 0.2. Then, the bacteria were inoculated into 96-well microtiter plate wells containing 200 μL of THB alone (untreated wells) or adding the tylosin solution with the final concentrations of 1/4 MIC (0.125 μ g/mL). In addition, a negative control (with THB alone) and a positive control (with bacteria alone) were also included. After incubation at 37°C for 72 h without shaking, the medium was removed by aspiration, and the wells were washed three times with sterile physiological saline. Biofilm cells were removed from wells by sonication for 5 min in 200 μL of THB. The cell suspensions (*n* = 3) underwent 10-fold dilutions in THB, and 100 μL of each dilution was spot plated onto THB soft-agar plates and incubated at 37°C for 24 h. All the experiments were performed in triplicate.

### Preparation of protein extracts

For biofilm cultures, *S. suis* was grown in THB in 100 mm polystyrene petri dishes at 37°C for 24 h. Then, the supernatant was removed and the dishes were washed twice with Tris-HCl buffer (50 mM, pH 7.5). The biofilms were detached by scraping. After being sonicated for 5 min (Bransonic 220; Branson Consolidated Ultrasonic Pvt. Ltd., Australia), the cells were centrifuged at 12,000 × g for 10 min at 4°C. Then, the cell pellets were washed twice with Tris-HCl buffer (Wang et al., 2012).

### Protein digestion and iTRAQ labeling

Protein digestion was performed according to the reported FASP procedure (Wisniewski et al., 2009). In brief, 200 μg of proteins at two different conditions (1/4 MIC of tylosin treated cells and nontreated cells) were added into 30 μL STD buffer (4% SDS, 100 mM DTT, 150 mM Tris-HCl pH 8.0) and ultrafiltered (Microcon units, 30 kD) with UA buffer (8 M Urea, 150 mM Tris-HCl pH 8.0). To block reduced cysteine residues, 100 μL 0.05 M iodoacetamide was added into UA buffer and incubated for 20 min in the dark. The filters were washed three times with 100 μL UA buffer and twice with 100 μL DS buffer (50 mM triethylammoniumbicarbonate at pH 8.5). Finally, the proteins were digested with 2 μg trypsin (Promega) in 40 μL DS buffer at 37°C for 16–18 h. Then, the resulting peptides were collected as a filtrate. The peptide content was estimated by UV light spectral density at 280 nm using an extinctions coefficient of 1.1 of 0.1% (g/l) solution calculated on the basis of the frequency of tryptophan and tyrosine in vertebrate proteins.

For the iTRAQ labeling, the peptides were labeled with the 8-plex iTRAQ reagent by following the manufacturer's instructions (Applied Biosystems). Each iTRAQ reagent was dissolved in 70 μL of ethanol and added to the respective peptide mixture. The peptides from the *S. suis* biofilms treated by tylosin were labeled with 115 isobaric reagent, and the peptides from the nontreated *S. suis* biofilms were labeled with 116 isobaric reagent. Then, the samples were multiplexed and vacuum dried. Three independent biological experiments were performed.

### Peptide fractionation with strong cation exchange (SCX) chromatography

SCX chromatography using the AKTA Purifier system (GE Healthcare) was used to fractionate the iTRAQ labeled peptides. After being reconstituted and acidified with 2 mL buffer A (10 mM KH_2_PO_4_ in 25% of ACN, pH 2.7), the peptides were loaded onto a PolySULFOETHYL 4.6 × 100 mm column (5 μm, 200 Å, PolyLC Inc., Maryland, U.S.A.). Then, the peptides were eluted at 1 ml/min with a gradient of 0–10% buffer B (500 mM KCl, 10 mM KH_2_PO_4_ in 25% of ACN, pH 2.7) for 2 min, 10–20% buffer B for 25 min, 20–45% buffer B for 5 min, and 50–100% buffer B for 5 min. The elution was monitored by absorbance at 214 nm, and the fractions were collected after every 1 min. The collected fractions (~30 fractions) were combined into 10 pools and desalted on C18 Cartridges [Empore™ SPE Cartridges C18 (standard density), bed I.D. 7 mm, volume 3 ml, Sigma]. Each pooled fraction was concentrated by vacuum centrifugation and reconstituted in 40 μl of 0.1% (v/v) trifluoroacetic acid and stored at −80°C for LC-MS/MS analysis.

### Liquid chromatography (LC) electrospray ionization (ESI) tandem Ms (MS/MS) analysis by Q exactive

Experiments were performed on a Q Exactive mass spectrometer that was coupled to Easy nLC (Thermo Fisher Scientific). A sample (10 μL) of each fraction was injected for the nano LC-MS/MS analysis. The peptide mixture (5 μg) was loaded onto a C18-reversed phase column (Thermo Scientific Easy Column, 10 cm long, 75 μm inner diameter, 3 μm resin) in buffer A (0.1% formic acid) and separated with a linear gradient of buffer B (80% acetonitrile and 0.1% formic acid) at 250 nl/min controlled by IntelliFlow technology for 140 min. MS data were acquired using a data-dependent top10 method dynamically choosing the most abundant precursor ions from the survey scan (300–1800 m/z) for HCD fragmentation. Determination of the target value was based on predictive Automatic Gain Control (pAGC). The dynamic exclusion duration was 60 s. Survey scans were acquired at a resolution of 70,000 at m/z 200, and the resolution for HCD spectra was set to 17,500 at m/z 200. The normalized collision energy was 30 eV, and the underfill ratio, which specifies the minimum percentage of the target value likely to be reached at maximum fill time, was defined as 0.1%. The instrument was run with the peptide recognition mode enabled.

### Sequence database searching and data analysis

The MS/MS spectra were searched using the MASCOT engine (Matrix Science, London, UK; version 2.2) in the Proteome Discoverer 1.3 (Thermo Electron, San Jose, USA.) against the Uniprot *S. suis* fasta database (38,369 sequences, downloaded March 4th, 2013) and the decoy database. False discovery rates (FDR) were calculated by running all spectra against a decoy database using the MASCOT software. To identify proteins, the following options were used: Peptide mass tolerance = 20 ppm, MS/MS tolerance = 0.1 Da, Enzyme = Trypsin, Missed cleavage = 2, Fixed modification: Carbamidomethyl (C), iTRAQ 8plex (K), iTRAQ 8plex (N-term), Variable modification: Oxidation (M). The quantification was performed based on the peak intensities of the reporter ions in the MS/MS spectra. The ratio of label 115 and 116 represents the expression of proteins with the protein identification confidence of a 1% FDR (Unwin et al., 2010). The proteins were considered over expressed when the iTRAQ ratio was above 1.5 and under expressed when the iTRAQ ratio was lower than 0.67. The Proteome Discoverer tool was used to categorize the proteins detected by Gene Ontology (GO) annotation according to the cellular component, biological process and molecular function.

## Results

### Effect of tylosin against biofilm formation *In vitro* by the TCP assay

**Figure 1:**
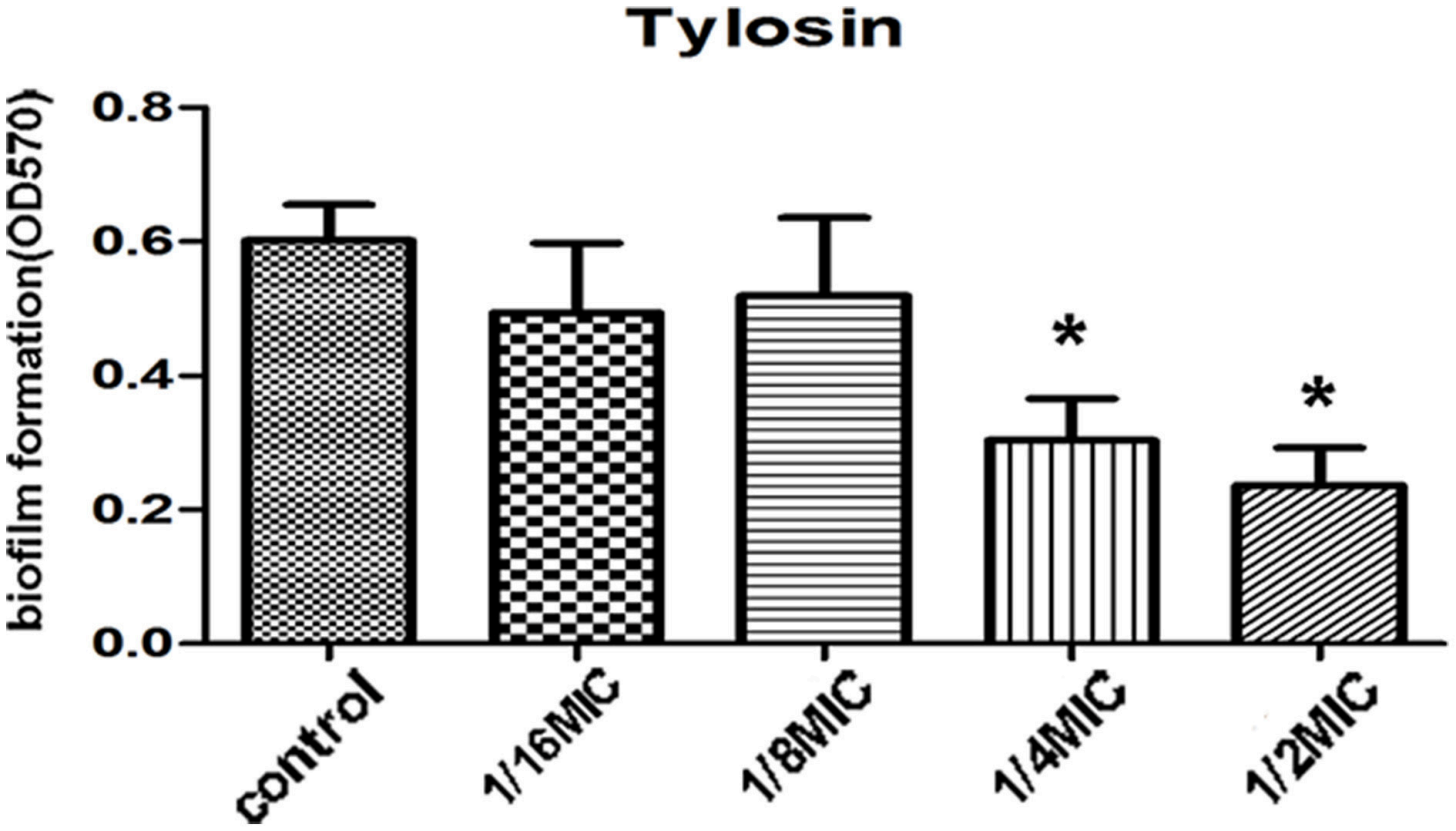
**Effect of tylosin at different concentrations on *S. suis* ATCC 700794 biofilm formation**. The data are expressed as the means ± standard deviations. Significant decrease (^*^p < 0.05) compared with control biofilm formation of *S. suis in vitro*.

We evaluated the action of tylosin on biofilm growth *in vitro*. The MIC against *S. suis* was 0.5 μg·mL^−1^. Tylosin at 1/2 MIC and 1/4 MIC caused a significantly higher reduction in the biofilm-forming ability of *S. suis* compared with positive control (*p* < 0.05). However, there was no pronounced effect for 1/16 MIC and 1/8 MIC of tylosin on biofilm formation of *S. suis* (*p* > 0.05; Figure 1).

### Direct observation of biofilm formation *In vitro* by sem

**Figure 2:**
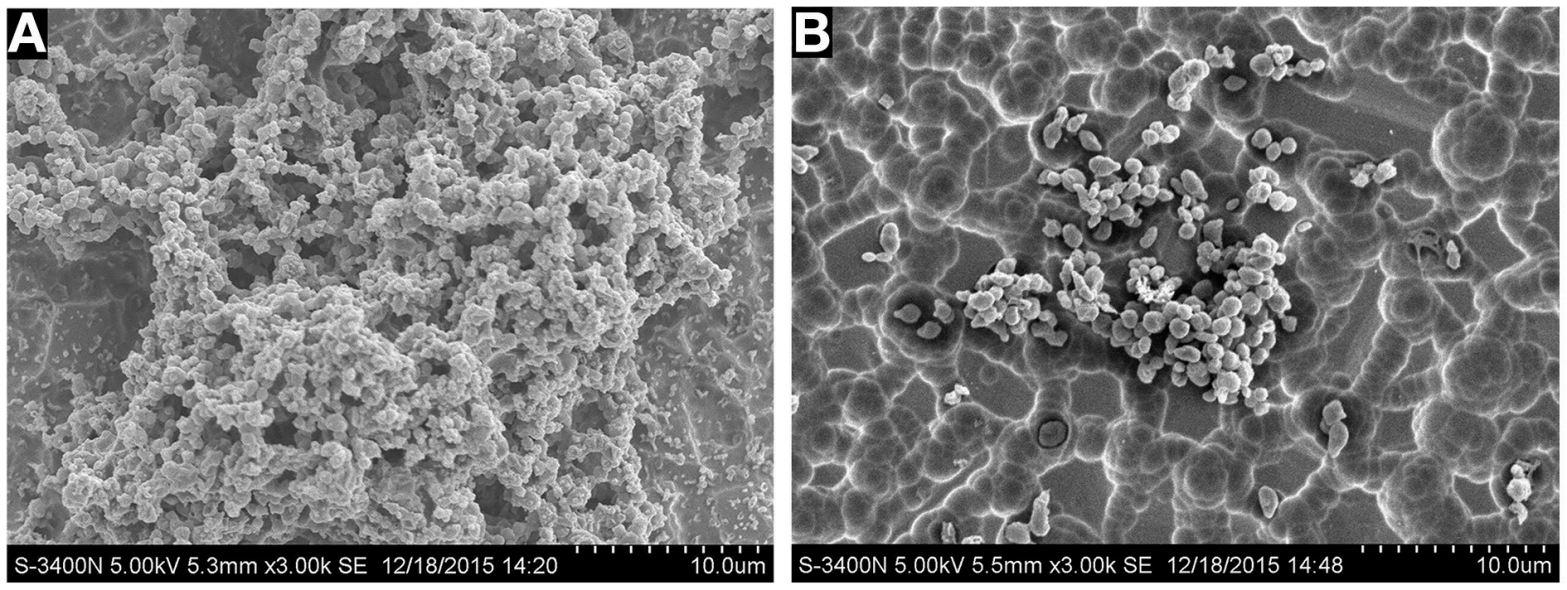
**(A)** Biofilm formation of *S. suis* without tylosin. **(B)** Biofilm formation of *S. suis* with 1/4 MIC tylosin treatment.

SEM analysis was performed to observe the 1/4 MIC of tylosin treated cells and nontreated cells biofilm formation by *S. suis* under same growth conditions. As shown in Figure 2A, the surface of the glass slide is almost entirely covered by the aggregates and microcolonies of *S. suis* when growth was carried out in the culture medium without tylosin. However, when the culture medium was added to 1/4 MIC of tylosin, the biofilms were characterized by the presence of small clusters of cells interspersed amongst individual cells (Figure 2B). This result showed that the biofilm formation of *S. suis* was inhibited by the tylosin of 1/4 MIC *in vitro.*

### Colony forming unit (CFU) enumeration

**Figure 3:**
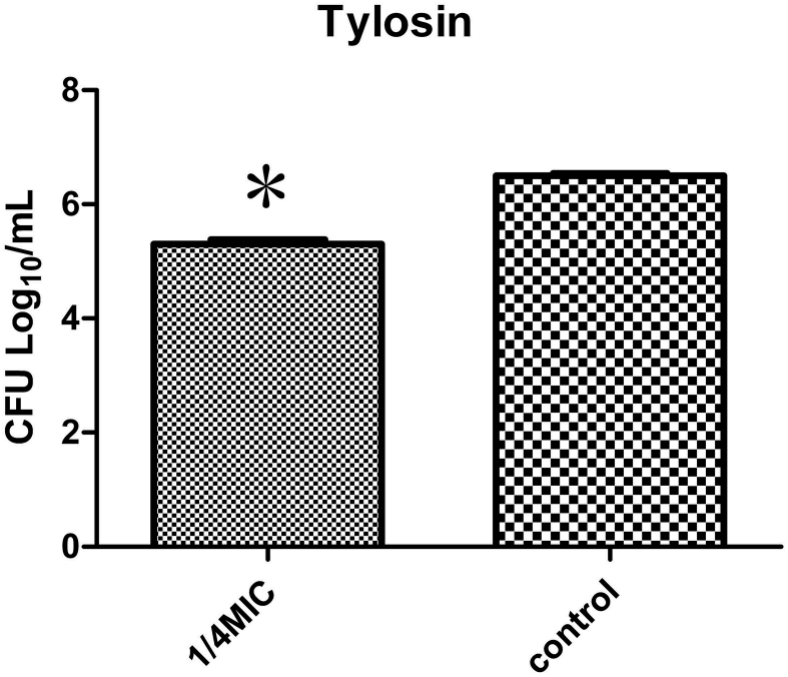
**Effect of 1/4 MIC of tylosin on *S. suis* ATCC 700794 biofilm formation by colony forming unit (CFU) enumeration**. The data are expressed as the means ± standard deviations. Significant decrease (^*^p < 0.05) compared with control biofilm formation of *S. suis in vitro*.

To better assess action of tylosin on biofilm quantitatively, the CFUs of *S. suis* were counted. The viability of *S. suis* treated with 1/4 MIC of tylosin was different from the viability of untreated *S. suis*. The number of CFUs/mL in treated biofilms (5.3 log_10_ CFUs/mL) was significantly fewer than in nontreated biofilms (6.5 log_10_ CFUs/mL; *p* < 0.05). The number of CFUs/mL in treated biofilms (5.3 log_10_ CFUs/mL) was significantly fewer than in nontreated biofilms (6.5 log_10_ CFUs/mL; *p* < 0.05) (Figure 3). The findings demonstrated that 1/4 MIC of tylosin remained effective in decreasing the viability of *S. suis*.

### Sub-MIC tylosin inhibits biofilm formation and differentially expressed proteins by iTRAQ

**Figure 4:**
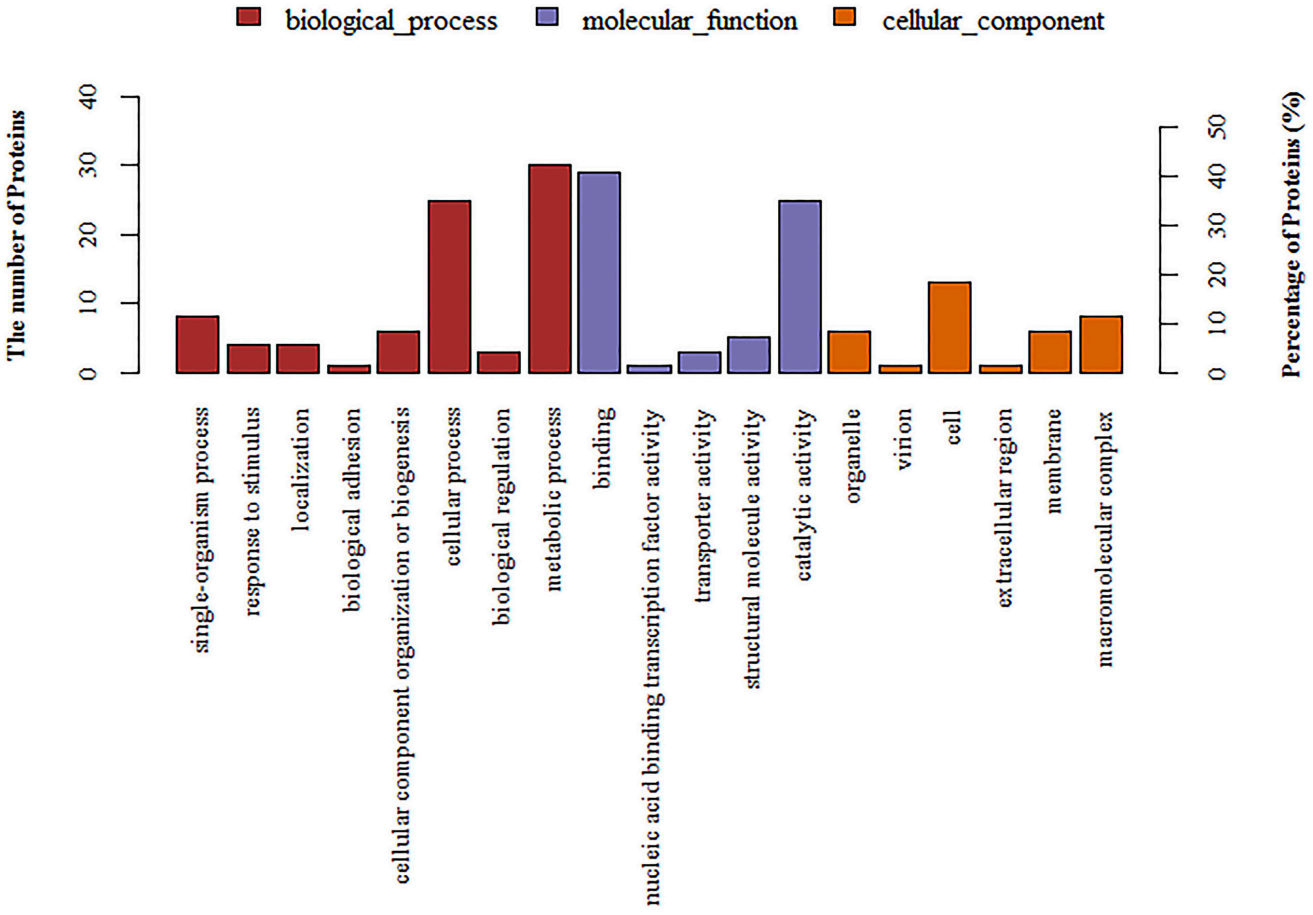
**Annotation of differentially regulated protein (ratio >1.5 or < 0.67) functions by Gene Ontology (GO)**.

A total of 1501 proteins were identified by iTRAQ. Detailed information is shown in the Supplementary File. A ratio of proteins with >1.5 or < 0.67 (*p* < 0.05) was considered to be differentially expressed. Based on this criterion, 96 differentially expressed proteins were identified in 1/4 MIC tylosin treated cells and nontreated cells. Detailed information could is shown Table 1. These proteins were detected by GO annotation. Thirty-five proteins were deleted from the database. The remaining 61 proteins were classified into biological process, molecular function and cellular component (Figure 4). The results regarding the biological process were as follows: single-organism process (8, 13%), response to stimulus (4, 7%), localization (4, 7%), biological adhesion (1, 2%), cellular component organization or biogenesis (6, 10%), cellular process (25, 41%), biological regulation (3, 5%), metabolic process (30, 49%). The results regarding molecular function were as follows: binding (29, 48%), nucleic acid binding transcription factor activity (1, 2%), transporter activity (3, 5%), structural molecule activity (5, 8%), catalytic activity (25, 41%). The results regarding cellular component were as follows: organelle (6, 10%), virion (1, 2%), cell (13, 21%), extracellular region (1, 2%), membrane (6, 10%), macromolecular complex (8, 13%).

**Table 1:** **List of differentially expressed proteins in tylosin treated cells**.

**Accession**	**Description**	**Fold change^a^**
G7SDH9	Putative uncharacterized protein	0.555777
A4W1G9	Phosphotransferase system cellobiose-specific component IIC	0.521547
G7SA24	Cold-shock DNA-binding domain protein	0.394546
F4EDP5	Putative uncharacterized protein	0.568016
R4NL31	MF3-like protein	0.63094
E9NQ29	CPS16V	0.452748
G5KZ73	NADH:flavin oxidoreductase/NADH oxidase family protein	0.459622
G7S4C2	Major membrane immunogen, membrane-anchored lipoprotein	0.643938
A8CUL3	Integrase	0.46773
G7SM99	Type I site-specific restriction-modification system, R (Restriction) subunit and related helicase	0.506277
G7SLJ0	Putative competence-damage inducible protein	0.564703
A4W1W3	F0F1-type ATP synthase, subunit a	0.586873
G7RZW0	Sugar ABC transporter permease	1.508325
G7S2T8	Alcohol dehydrogenase	0.610195
G7SDG9	Laminin binding protein	1.524593
R4NLJ6	Oligopeptide ABC transporter, periplasmic oligopeptide-binding protein OppA	0.644177
G7S371	Fructose-bisphosphate aldolase	0.623705
M1UGE2	Uncharacterized protein	0.647168
A4VZM2	Cell cycle protein GpsB	0.631018
A4W2D2	Cation transport ATPase	1.676356
G5L1V6	50S ribosomal protein L9	0.6638821
G7SEP2	Putative uncharacterized protein	1.502566
A4VVZ3	tRNA dimethylallyltransferase	1.510883
G7S5B8	3-isopropylmalate dehydrogenase	1.513117
G7SA68	UDP-N-acetylmuramate–alanine ligase	0.656503
G5L098	Transcriptional regulator Spx	1.514636
E8UKC3	Uncharacterized protein	1.507837
G7SFU4	Putative uncharacterized protein	1.543591
A4VV99	DNA repair protein	1.545594
A4W2Z7	FMN-dependent NADH-azoreductase	1.527496
F4EC05	Putative uncharacterized protein	1.57743
C6GNL8	30S ribosomal protein S21	1.545471
K7ZNG8	DNA recombination/repair protein (Fragment)	1.558023
G7S3K5	ABC-type cobalt transport system, ATPase component	1.583854
G7SM56	Ribosomal RNA small subunit methyltransferase H	1.594936
A2VC24	Muramidase-released protein	1.579304
A4W3C1	Putative phosphotyrosine protein phosphatase	1.60975
G7S4U0	Putative uncharacterized protein	1.596783
G7SP92	Putative uncharacterized protein	1.658448
G7S7E3	Helicase	1.516925
M1VK55	Glycosyltransferase	1.662795
G7S3P9	Putative uncharacterized protein	1.673475
A4W106	Signal transduction histidine kinase	1.62492
B0M0G7	Phosphomethylpyrimidine kinase (Fragment)	1.633436
A5JSJ7	Putative uncharacterized protein	1.681661
G5L226	Type III restriction-modification system, restriction endonuclease subunit	1.697991
G7S7K4	Putative uncharacterized protein	1.645719
B9WY95	Peptidase M20	1.647536
G7S4T2	Branched-chain alpha-keto acid dehydrogenase subunit E2	1.673032
G5KX48	ABC-type uncharacterized transport system, permease component	1.705109
G7S178	Putative uncharacterized protein	1.774886
M1VDL7	ABC transporter permease protein	1.942183
G7S6Z3	Putative uncharacterized protein	1.956584
J7KIA5	Abortive infection bacteriophage resistance related protein	1.999106
G7SJZ7	Primosomal protein N'	1.720079
G7SHW7	Putative uncharacterized protein	2.001559
Q9EZW2	Elongation factor Tu (Fragment)	1.739971
A4VVJ5	Uncharacterized protein	2.141695
M1VJZ8	Zeta toxin	1.74309
G5L2V0	NADPH:quinone reductase and related Zn-dependent oxidoreductase	1.79706
R4NU43	Aromatic amino acid aminotransferase gamma @ N-acetyl-L,L-diaminopimelate aminotransferase	2.144605
A4W296	Uncharacterized protein	1.837367
G7SKQ0	CHAP domain containing protein	2.179882
B9WVD6	Putative uncharacterized protein (Fragment)	2.226792
G5L2N5	L-fucose isomerase	1.859887
B9WTA1	Putative uncharacterized protein	2.290848
B9WXU6	ABC transporter related protein	1.873566
G8DU82	Transposase	2.376768
G7S242	Putative ABC transporter	1.883471
A4VZ91	50S ribosomal protein L32	1.915877
G7SDX6	50S ribosomal protein L7/L12	1.918196
G5KZ86	Phosphatidylserine/phosphatidylglycerophosphate/ cardiolipin synthase-like protein	1.965488
G7SIQ5	Putative uncharacterized protein	2.451703
G7S7J2	Putative uncharacterized protein	2.481527
G7SPA1	Putative scaffolding protein	2.584249
G7S7A9	FAD-dependent pyridine nucleotide-disulfide oxidoreductase	1.970356
G7S8Q2	Peptidase M22 glycoprotease	1.978772
M1TIM7	Methylated DNA-protein cysteine methyltransferase	2.161583
G7SBK2	Elongation factor Ts	2.266517
M1VE47	Fic/DOC family protein	2.606274
M1UFP9	Thiamine-phosphate synthase	2.298028
G7S535	Fructose-6-phosphate aldolase	2.553516
G5L351	NsuB	2.694988
B9WUV5	Transcriptional regulator, DeoR family	2.65073
R4NW55	Plasmid replication protein Rep and AAA-class ATPase domain protein	2.779765
G7RZ18	Sortase-like protein	2.791504
S6B433	RecN protein (Fragment)	3.007243
G5KZR3	Glutathione S-transferase	3.262393
R4NLK5	SSU ribosomal protein S1p	3.46851
R4NWB6	Uncharacterized protein	8.306942
G5L259	NADP-dependent glyceraldehyde-3-phosphate dehydrogenase, putative	2.781098
D5AFN4	Xaa-Pro dipeptidyl-peptidase	3.095053
G7SMG3	Elongation factor G	3.667679
G7SD52	ABC superfamily ATP binding cassette transporter, membrane protein	3.201581
G5L1N9	Nucleoid DNA-binding protein	3.796473
G7S8P5	Phosphoglycerate kinase	11.34908

*Fold change^a^, the ratio of different expression level between Sub-MIC tylosin treated cells and nontreated cells*.

## Discussion

A TCP assay is based on the ability of bacteria to form biofilms on the bottom of tissue culture plates and is mainly used to identify the formation of bacterial biofilms (Mathur et al., 2006; Okajima et al., 2006; Presterl et al., 2007). Bacteria are grown in cell culture plates, and the quantity of a biofilm is detected by staining the wells with crystal violet according to the correlation between OD values and biofilm formation (Mathur et al., 2006).

To further confirm *S. suis* biofilm formation, the structure of the *S. suis* biofilm was identified by scanning electron microscopy (SEM). The biofilm examination relied heavily on SEM. Because of its high magnification, the biofilm microstructure is observed clearly. These findings further confirm that *S. suis* can form biofilms *in vitro*. Although these procedures have their advantages, the TCP procedure lacks specificity and sensitivity because crystal violet stains all of the components of the biofilm and could potentially stain non-biofilm material. More specific and sensitive techniques can be performed to assess each of the individual components. This method is good for screening purposes.

Tylosin is a frequently used drug for the treatment of *S. suis* infection. Upon treatment with this antimicrobial agent, *S. suis* is inevitably exposed to a sub-inhibitory level of the agent. Therefore, we studied its effect in sub-inhibitory concentrations on *S. suis* biofilm formation. Tylosin can inhibit *S. suis* biofilm formation at sub-inhibitory concentrations in a dose-dependent manner. In addition, the inhibition of biofilm formation varied among the antimicrobial agents. Numerous reports have showed biofilm formation in the presence of sub-inhibitory concentrations of antimicrobial agents (Dunne, 1990; Carsenti-Etesse et al., 1993; Rupp and Hamer, 1998; Rachid et al., 2000; Yang et al., 2015). Our results are in agreement with these previous findings. Our results showed a better activity of tylosin against *S. suis* biofilm formation. However, further studies should be conducted to confirm and clarify the relationship between the relative adherence-inhibiting properties of tylosin and their mechanisms. These results may provide information regarding the clinical use of antimicrobial agents against biofilm-forming bacteria.

Our data identified proteins related to biofilm growth that have previously been uncharacterized. iTRAQ analyses showed that the regulation of metabolism plays a key role during *S. suis* biofilm growth. First, the iTRAQ quantitative data revealed that carbohydrate metabolism may be particularly important during *S. suis* biofilm growth. It was reported that fructose bisphosphate aldolase and glyceraldehyde-3-phosphate dehydrogenase were significantly decreased in *Streptococcus pneumoniae* biofilms (Allan et al., 2014). In our study, there was a >two-fold increase of glyceraldehyde-3-phosphate dehydrogenase in the tylosin inhibiting biofilm formation. In addition, the levels of fructose bisphosphate aldolase (fold change: 0.62) were down-regulated. It was reported that phosphoglycerate kinase was up-regulated in *S. suis* biofilms compared with planktonic cells by comparative proteomic analysis (Wang et al., 2012). Similarly, biofilms of *Pseudomonas aeruginosa* (Sauer et al., 2002) and *Staphylococcus xylosu*s (Planchon et al., 2009) exhibit up-regulated phosphoglycerate kinase. There was a >11-fold increase in the level of phosphoglycerate kinase in the tylosin inhibiting biofilm formation. Phosphoglycerate kinase is a glycolytic enzyme that functions in the conversion of glyceraldehyde 3-phosphate into 1, 3-diphosphoglycerate. Glycolysis may play a pivotal role during *S. suis* biofilm growth. Furthermore, histidine metabolism may play an important role in biofilm formation. Histidine kinase (fold change: 1.62) was up-regulated in the tylosin-inhibited biofilm formation. Compared with other amino acids, L-His had the strongest effect on biofilm induction (Cabral et al., 2011). Histidine metabolism is also found to be involved in biofilm formation and is confirmed by gene disruption (Cabral et al., 2011). Histidine kinase is an important signaling molecule in biofilm formation in gram-positive and negative bacteria (McLoon et al., 2011; Shemesh and Chai, 2013; Yang et al., 2014; Grau et al., 2015). In addition, several ABC transporter system proteins were significantly up-regulated in the tylosin inhibiting biofilm formation. This finding is consistent with the previous results of our laboratory showing that sub-MIC erythromycin inhibits *S. suis* biofilm formation (Zhao et al., 2015). The carbohydrate substrate selection and fermentation determine ABC transporter proteins were significantly up-regulated in tylosin-treated cells, suggesting that *S. suis* had the capability of metabolizing a wide range of carbohydrates during biofilm development (Hardy et al., 2001; Marion et al., 2011; Bidossi et al., 2012; Haertel et al., 2012). Moreover, ABC transporters are important because they regulate respiration and biofilm formation, which in turn affect the rate of electricity production and bioremediation (Selvaraj et al., 2014). Furthermore, in the mutants of a *pneumococcal* biofilm screen, ABC transporters were shown to be defective in colonization (Munoz-Elias et al., 2008).

Cell surface proteins play a crucial role in biofilms. Within the biofilm, bacterial cells are embedded in a self-produced extracellular matrix. This matrix protects bacteria against a number of environmental insults. However, the matrix also confines bacterial access to fresh nutrients. Thus, the verified increase in the expression of transmembrane channels appears to be an essential requirement for the entrance of important nutrient-containing fluid (Costerton et al., 1994). In addition to acting, as channels, porins may act as potential targets for adhesion to other cells and may mediate cell attachment through binding to the proteins released for biofilm formation (Dallo et al., 2010). The outer membrane proteins may mediate cell attachment through binding to the released proteins for biofilm formation. Membrane proteins such as OmpA mediate cell adhesion in *Acinetobacter baumannii* (Dallo et al., 2010). The key developmental stages of biofilm development have been reported to be adhesion to surfaces, aggregation of micro colonies, and further expansion of the microbial community. The putative involvement of many genes encoding large cell surface proteins is adhesion to the epithelium and biofilm formation (Pridmore et al., 2004; Walter et al., 2005; Frese et al., 2011). Pham (Pham et al., 2010) found increased expression of outer membrane proteins in the *Tannerella forsythia* biofilm cells by using quantitative non-gel-based proteomic techniques. In our study, the expression of membrane-anchored lipoprotein, phosphoglycerate kinase, sugar ABC transporter permease, ABC superfamily ATP binding cassette transporter membrane protein and L-fucose isomerase changed; these proteins belong to membrane proteins and cell-surface proteins and might be involved in some molecular functions including catalytic activity, motor activity, nucleotide binding, protein binding and transporter activity. For example, phosphoglycerate kinase is a *S. suis* surface protein that promotes cell adhesion and plays a key role in bacterial infection and invasion (Brassard et al., 2004; Wang and Lu, 2007). We predicted that these membrane proteins might affect the bacterial cell-cell interaction. Thus, membrane proteins might play a significant role in biofilm formation.

*S. suis* virulence proteins related to infection, persistence and competitive fitness were mostly down regulated when sub-MIC tylosin was used to inhibit biofilm formation. There was a >1.5-fold decrease in the level of NADH oxidase. NADH oxidase regulates competence, virulence, and pneumococcal persistence by its actions as an oxygen sensor, in detoxifying oxygen, and in increasing the efficiency of glucose breakdown (Auzat et al., 1999) and plays an important role in *pneumococcal* infection in animal models of pneumonia (Yu et al., 2001). NADH oxidase is encoded by *nox*. The virulence and persistence in mice of a blood isolate was attenuated by a *nox* insertion mutation (Auzat et al., 1999). Thus, this protein appears to be of major importance in the growth of biofilms.

## Conclusions

Our study suggested that sub-MICs of tylosin could inhibit *S. suis* biofilm formation *in vitro*. We used a robust and reliable comparative proteomic technique (iTRAQ) to compare the abundances of proteins from 1/4 MIC of tylosin treated cells and nontreated cells. Finally, in sub-MIC tylosin inhibiting cells, we identified 96 differentially expressed proteins when the protein had a fold-change of more than a ratio >1.5 or < 0.67 (*p* < 0.05). Our proteomic data suggested general changes in metabolism (such as phosphoglycerate kinase) and surface proteins (such as ABC transporter proteins) involved in biofilm formation. Overall, our results indicated that *S. suis* metabolic regulation, cell-surface proteins, and virulence proteins appear to be of importance in biofilm growth by sub-MIC tylosin treatment. Thus, our data revealed the rough regulation of biofilm formation that might potentially be utilized to manage biofilm infections of *S. suis*.

## Author contributions

SW the design whole experiment. YL directed the completion of the experiment. YY, YZhao, HZ, JB, JC, YZhou, CW provided help during the experiment.

### Conflict of interest statement

The authors declare that the research was conducted in the absence of any commercial or financial relationships that could be construed as a potential conflict of interest.
